# Case report: Beneficial long-term effect of the atrial-flow-regulator device in a pediatric patient with idiopathic pulmonary arterial hypertension and recurring syncope

**DOI:** 10.3389/fcvm.2023.1197985

**Published:** 2023-09-08

**Authors:** Joseph Pattathu, Sebastian Michel, Anja Ingrid Tengler, Guido Mandilaras, Andre Jakob, Robert Dalla Pozza, Nikolaus A. Haas

**Affiliations:** Division of Pediatric Cardiology and Intensive care, University Hospital, Ludwigs-Maximilians University Munich, Munich, Germany

**Keywords:** pulmonary arterial hypertension, cardiac catherization intervention, atrial septostomy, atrial flow regulator (AFR) - device, pediatric cardiology

## Abstract

We report the long-term effect after successfully implanting an 8 mm Atrial-flow-regulator (AFR) device in a 7-year-old girl with idiopathic pulmonary hypertension with persistent syncope under triple therapy with significant improvement after implantation and absence of any further syncope. Early Implantation of the AFR device (Occlutech, Germany) can be efficient and safe interventional therapy option for pulmonary arterial hypertension with a history of syncope.

## 1. Introduction

**Figure 1:**
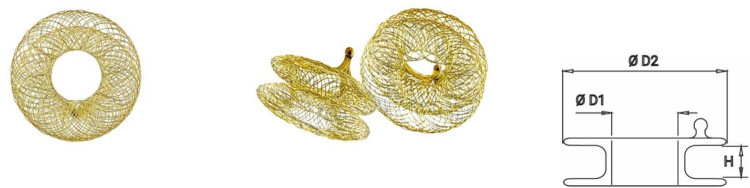
AFR device. Adapted with permission from Occlutech (Germany). AFR device. Adapted with permission from Occlutech (Germany) unfolded, D1, Inner circumference; D2, outer circumference; H, height.

Pulmonary arterial hypertension (PAH) with childhood-onset is a serious and scarce disease with a desolating prognosis (1). The range of the aetiologies in pediatric PH is very different to those in adults, with a higher prevalence of idiopathic arterial hypertension (IPAH), and pulmonary arterial hypertension in the context of congenital heart disease (PAH-CHD), as well as in the context of developmental lung disease. The multitude of aetiology, phenotype, and prognosis requires a tailored approach in children (2). Patients of any age group initially show non-specific symptoms such as fatigue, failure to thrive, dyspnoea, and even syncope (3). Syncope, the most serve sign in PAH, reflects a significant reduction of transpulmonary blood flow during acute pulmonary hypertension crisis, which occurs late compared to grown-ups (1). The 5-year survival under the latest treatment options rate is around 75% (2). In case of further disease progression targeted pharmacological treatment options such as calcium-channel blockers, endothelin-receptor antagonists (ERA), phosphodiesterase-5 inhibitors (PDE-5 Inhibitors) and prostacyclin analogues are required in these patients (1, 2). Especially in this stadium of the disease, these patients experience low-cardiac output, probably confirmed by the occurrence of syncope (3, 4). Once syncope occurs, improvement of clinical symptoms can be provided by performing a balloon-atrial-septostomy (BAS), as the evidence shows in adults. However, after BAS the long-term outcome is unfortunate, significantly if the right atrial pressures are elevated due to the uncontrolled size of the BAS. Subsequent acute and severe desaturation can occur (5). A concise shunt size might reduce this risk. The AFR (Occlutech, Germany) (Figure 1) could demonstrate promising data in terms of the long-term outcome of adult patients in this defined condition (6).

### 1.1. Ethical statement

The authors are responsible for all parts of the work to securing that aspect of accuracy, or integrity is thoroughly investigated and resolved. The patient's integrity was ensured with the Helsinki Declaration (as revised in 2013). Written informed consent was obtained from the patient to publish this case report and accompanying images. A copy of the written consent is available for review by the editorial office of this journal.

### 1.2. Patient information and clinical findings

In our case, the implantation of an eight-millimetre AFR device (Occlutech, Germany) was performed in a 7-year-old girl with idiopathic PAH. The girl was referred after having a confirmed Mycoplasma pneumonia infection only with fever, missing other symptoms like coughing or dyspnoea. However, after recovering from the infection, she presented to the pediatric cardiologist at the local hospital with decreased exercise capacity and shortness of breath. A significant dilatation of the right atrium, the right ventricle, and the pulmonary artery could be detected by echocardiography. The estimated right ventricular pressure was around 50 mmHg, and the pro-BNP was 8,683 pg/ml without congenital heart disease. The ECG showed sinus tachycardia with 120 bpm and a p-pulmonale.

## 2. Diagnostic assessment

An extensive evaluation of pulmonary hypertension was initiated. Nevertheless, except for positive Mycoplasma pneumonia result, the CT scan, coagulation disorder screening, allergy diagnostic, autoimmune-diagnostic, lung function test, ultrasound of the abdomen, polysomnography, EEG and Genetic testing showed no secondary cause for the PAH. The first cardiac catheterisation (Table 1) showed no severe PAH (PAPm 25 mmHg, PCW 6 mmHg, PVR 3.8 WU, CI 4.8 L/min/m^2^). Clinical symptoms improved, and the 6-minute walking test and the NT-pro-BNP reached a normal range (i.e., 684 m).

**Table 1:** Cardiac catheterisation data.

Date	Condition	mPAP (mmHg)	PCW (mmHg)	PVR (Wood units)	CI (L/min/m^2^)
30/06/2014	baseline	24	6	3.8	4.8
09/01/2015	baseline	25	15	2.2	4.48
02/05/2016	Bef. AFR	44	3	6.65	6.22
	Aft. AFR	28			
16/04/2019	baseline	28	6	3.94	5.83
	Oxygen	31	8	3.53	8.22

mPAP, mean pulmonary arterial pressure; PCW, wedge pressure; PVR, pulmonary vascular resistance; CI, cardiac index, in yellow data at the time of implantation.

Due to the positive mycoplasma serology, the further pneumology evaluation showed suspicious bronchiolitis obliterans in the CT scan, and the bronchoscopy showed moderate stenosis of the middle lung lobe. Further, the RV systolic pressure increased to approximately 70 mmHg, and targeted therapy with PDE-5 Inhibitors was initiated. A further rise of the RV systolic pressure to 90 mmHg occurred, and therapy was escalated with endothelin-receptor antagonists and inhaled Prostacyclin. Despite this regime, a further deterioration could be observed, and the girl developed recurring syncope under physical exercise (running to the schoolbus). She was unable to ride a bicycle or even to attend school. She woke up regularly at night hyperventilating, and her 6-minute walking test was less than 100 m then. The echocardiography showed supra-systemic pressure in the right ventricle (111 mmHg).

## 3. Therapeutic intervention

The initiation of intravenous Prostacyclin was discussed, but due to the recurring syncope, the balloon-atrial-septostomy + AFR implantation was performed.

**Figure 2:**
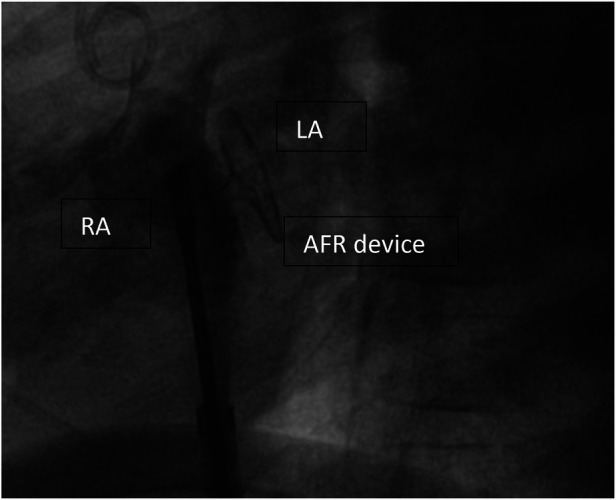
Implanted AFR device (Occlutech, Germany) before disconnecting. AFR device (Occlutech, Germany) right before deployment, RA, right atrium; LA, left atrium.

**Figure 3:**
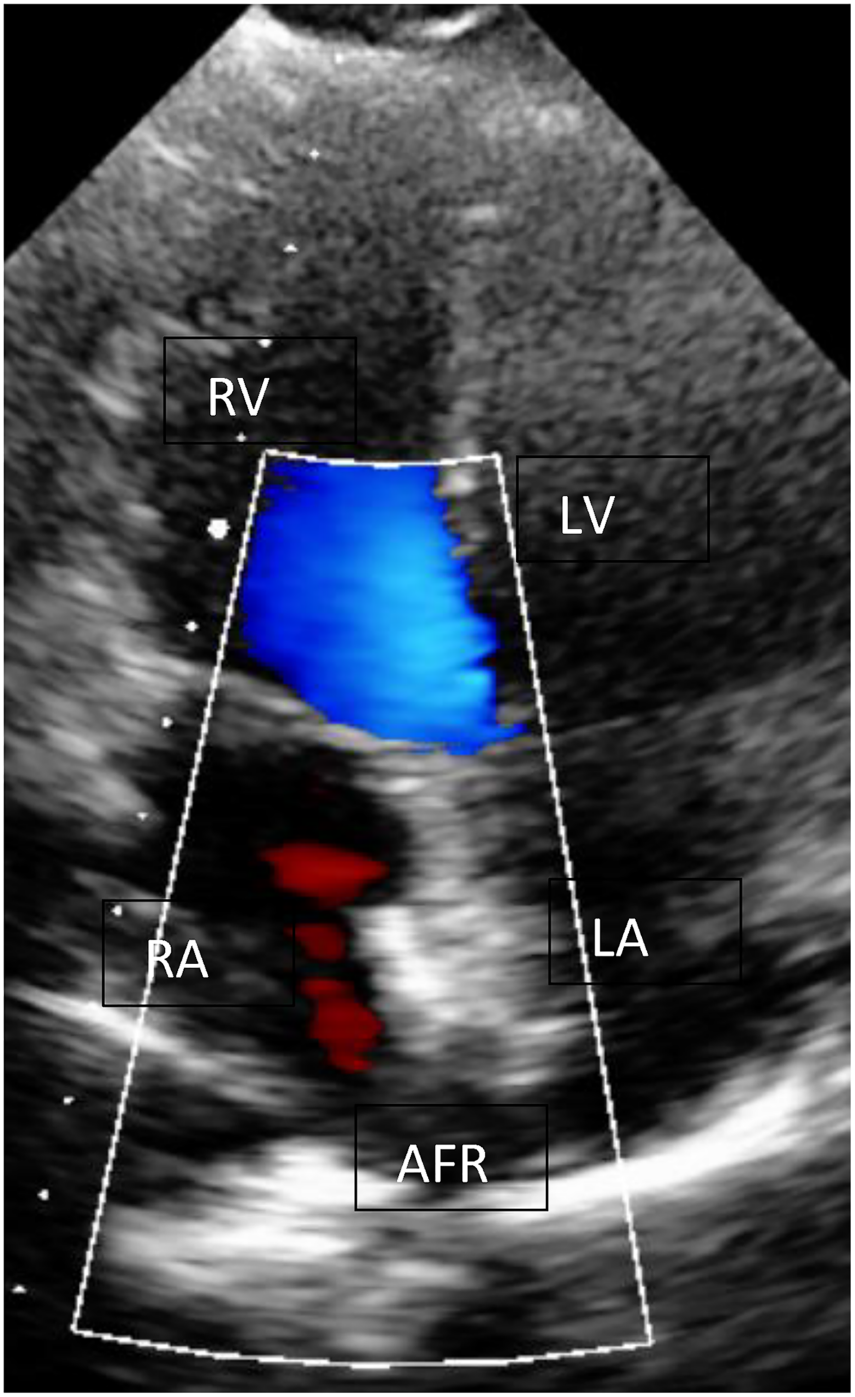
AFR device (Occlutech, Germany) in echocardiography. AFR device (Occlutech, Germany) with L-R shunt at rest; RA, right atrium; LA, left atrium; LV, left ventricle; RV, right ventricle.

Due to the severity of the condition and the novel device, which was not CE approved then, we obtained informed consent, and the Ethical committee approved the off-label use of an 8 mm AFR device. We performed the cardiac catheterisation under sedation. During induction, the child developed a PH crisis, which could be treated with Catecholamines and intravenous Prostacyclin. However, the second episode of a PH crisis occurred during device implantation. This time required a short period of resuscitation. Under the application of Adrenalin i.v. and Prostacyclin directly into the pulmonary artery, a stabilisation of the condition could be established. We perforated the intra-atrial septum with a Brockenorough transseptal puncture needle under the guidance of transesophageal echocardiography (TEE), the puncture hole was dilated, and a 12 Fr sheath was inserted over the interatrial communication into the left atrium so that 8 mm AFR device could be deployed (Figure 2). After confirming the correct position and checking the oxygen saturation level of greater than 85% and stable hemodynamic parameters, the device was completely and successfully released. The postinterventional echocardiography confirmed a right-to-left colour flow through the 8 mm atrial communication (Figure 3). After the intervention, anticoagulation with ASS was established.

## 4. Follow-up and outcomes

**Figure 4:**
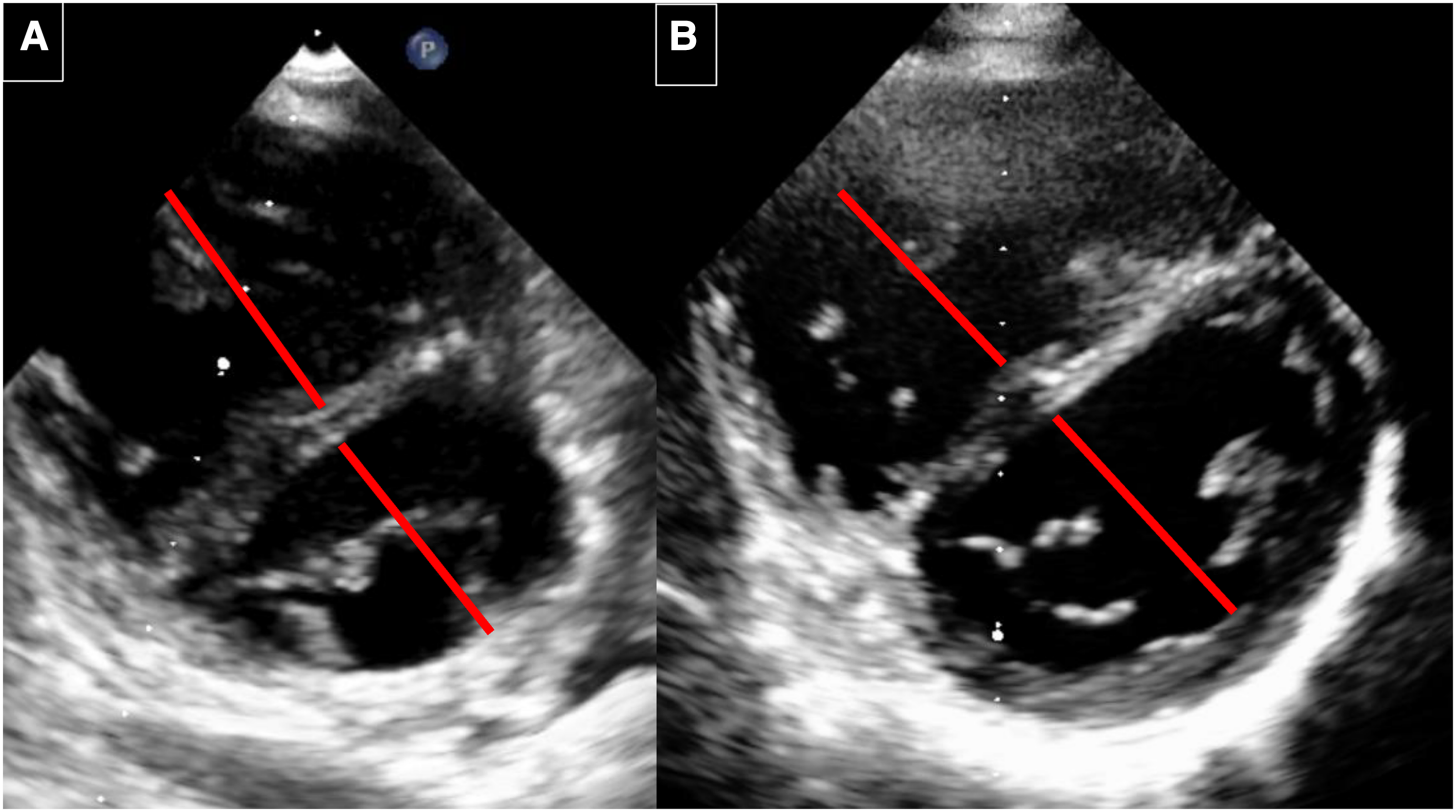
Echocardiography before and 6 years after implantation. (**A**) Before implantation: RV with septal flattening before AFR implantation. The RV/LV diameter ratio is 1: 0.87. (**B**) 6 years after AFR implantation. RV with decrease of the RV volume and improved LV filling. The RV/LV ratio is 0.87/1.

After the AFR device (Occlutech, Germany) implantation, she recovered within 1 day and never developed syncope again. In the following two years, a normalisation of the saturation over 95% could be observed, her NT–pro BNP levels went back to normal values, and her exercise capacity increased. The saturation drops to around 75% under exercise conditions. The cardiac catheterisation 3 years after AFR device implantation showed significant improvement (Table 1). 6 years after device implantation, her baseline saturation remained normal (98%, NHYA functional class II), the right ventricular volume and size decreased (Figure 4), NT–pro-BNP values were satisfying (Table 2) and the 6-minute walking distance was around 540 m.

**Table 2:** NT-pro BNP values before and after AFR implantation.

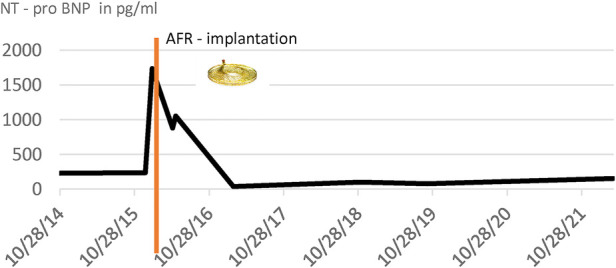

Elevated NT – pro BNP levels at the time of implantation, Red line: time of AFR – Implantation, normalization of NT – pro BNP levels after implantation, and maintaining levels within a normal range over a long-term period.

## 5. Discussion

Idiopathic PAH in children follows a more aggressive course than adults, so children with PAH suffer more from syncope (7, 8). In pediatric patients with PAH, the aetiology for syncope is more related to the acute impairment of cardiac output. As the pulmonary vascular resistance is acute and severely increasing, the result is a reduction of the transpulmonary blood flow with significant dilation of the RV and, as a consequence, underfilling of the LV, resulting in a low stroke volume and low blood pressure due to a decrease in cardiac output (7). At the time of referral, the child presented a diverse risk profile according to the pediatric risk stratification tables (9). Under specific pharmacological triple therapy with inhaled Prostacyclin, the girl maintained at NHYA functional class III with persisting syncope. Due to the multitude of challenges, such as providing sufficient support in the hometown, the significant side effects of Prostacyclin i.v., risk of catheter infection, in this case, intravenous Prostacyclins weren't added (1). According to the pediatric treatment algorithm, a permanent atrial septum fenestration was considered since no further conservative therapy options seemed to be sensible and the a significant impairment in the quality of life was present (2).

In adults with end-stage PAH, Atrial septostomy might be an option as a bridge-to-transplant or destination therapy if targeted therapy is not well tolerated or the option for transplantation is restricted. Recently, it was elucidated that permanent atrial septostomy with the AFR device (Occlutech, Germany) significantly reduced the symptoms of syncope (10, 11). During a pulmonary hypertension crisis with syncope, the specific and well-dosed interatrial communication will provide systemic cardiac output and left ventricular filling through the right-to-left shunt at the cost of moderate desaturation. Indubitable, the shunt volume will determine the systemic oxygen saturation. Accordingly, allocating the suitable size of the device for implantation is essential (11). The oxygen saturation before the procedure should not be below 85%, and RA pressure should not be above 20 mmHg to enable safe device implantation. Previous data showed significant impairment in these patients' outcomes, especially with uncontrolled BAS (9, 11).

For the creation of interatrial communication, different techniques are practised. So far, the ESC guidelines for adult PAH patients recommend a graded balloon atrial septostomy technique (1). By applying this technique, the sequential dilatation of the created communication between the left and the right atrium reduces the risk of overshunting. This technique supports beneficial hemodynamic outcomes in thoroughly selected PAH patients (12).

A major issue is spontaneous closure in nearly 25% of the patients who have received an atrial septal defect (12). Consequently, techniques have been provided, such as tailored fenestrations in commercially available ASD devices, experimental stent implantations in different variations, and different techniques of delivery (12). The risk of these techniques are the uncertain size of the fenestrations, spontaneous closure, embolisation of the implanted stent, and serious cyanosis caused by overshunting (11, 13, 14).

A fenestration diameter of 8 or 10 mm for the AFR device (Occlutech, Sweden) is since 2019 authorised in adults with chronic left heart failure and drug-resistant, severe PAH. The controlled interatrial right-to-left blood flow allows decompressing of the right ventricle. It improves the cardiac output in the absence of relevant systemic desaturation implied the device is allocated assiduously for the patient (14). The fenestrated device with the self-expandable double-disc is available with 4, 6, 8, and 10 mm fenestrations. As the implantation of the AFR device (Occlutech, Germany) is effortless without major complications confirmed by Clinical trials, the AFR device (Occlutech, Germany) is a secure option (15, 16).

Interventional closure of Atrial septal defects with double disc devices is a routine and secure procedure in children unaffected by pulmonary hypertension. The AFR device (Occlutech, Germany) implantation is comparable to that (14, 17). In adults, a positive long-term outcome could be demonstrated, showing a significant improvement in the cardiac index, a very low rate of device occlusions, and resolving the symptom of syncope without relapse (15, 16). More data is required through clinical trials with higher patient numbers and longer follow-up periods, but the results so far are ensuring.

However, limited experience is present in the pediatric cohort with just a small number of case reports and series describing pediatric patients with heterogenous etiologies of PAH (14, 18). In this case, an AFR device (Occlutech, Germany) with an 8 mm fenestration could be successfully implanted in a 6-year-old child. The oxygen saturation was within a satisfying range and the absence of significant clinical symptoms for over 5 years is a tremendous outcome for this patient's devastating situation initially.

## 6. Conclusion

The AFR device (Occlutech, Germany) is a secure and effective interventional measure in adults with drug-resistant severe pulmonary hypertension, especially in those showing recurrent syncope. The AFR device is superior to the balloon-atrial septostomy in terms of safety and long-term outcome. This case shows again that even in smaller children, the implantation of an AFR device (Occlutech, Germany) is a very promising treatment option, which is also feasible in children. The 8 mm device also seems possible in terms of growth and adolescence, so in our case, platelet aggregation inhibitors were sufficient to maintain the shunt connection long-term. Nearly six years after implantation, exercise capacity is excellent, NT-pro BNP levels and oxygen saturation are within the normal range, and the shunt connection remains open. However, it seems crucial to consider early implantation at the occurrence of the first syncope or when deterioration of the PAH necessitates the escalation of pharmacological therapy to triple therapy. The early implantation will enable patients to accommodate the potential desaturation over time. Further investigations and clinical trials are required for the more widespread use of this therapeutic optimisation in PAH management.

## 7. Patients perspective

The implantation of the AFR device (Occlutech, Germany) was an absolute game changer. This enabled me to return to life and participate in school and limited exercise. To conclude, it facilitated reintegration with my peers.

## Data Availability

The original contributions presented in the study are included in the article/Supplementary Material, further inquiries can be directed to the corresponding author.
